# Case Report: Afatinib-Induced Interstitial Pneumonia: Experiences and Lessons From Two Patients

**DOI:** 10.3389/fphar.2021.698447

**Published:** 2021-10-13

**Authors:** Xiao Liu, Baozhen Ma, Tiepeng Li, Lingdi Zhao

**Affiliations:** ^1^ Department of Radiotherapy, Affiliated Cancer Hospital of Zhengzhou University and Henan Cancer Hospital, Zhengzhou, China; ^2^ Department of Immunotherapy, Affiliated Cancer Hospital of Zhengzhou University and Henan Cancer Hospital, Zhengzhou, China

**Keywords:** afatinib, interstitial pneumonia, glucocorticoid, lessons, lung cancer

## Abstract

**Background:** Afatinib has shown good efficacy in patients harboring uncommon *EGFR* mutations, but the incidence of afatinib-induced interstitial pneumonia should be alert as its rapid progression. Here, we report two cases of interstitial pneumonia during afatinib treatment.

**Case presentation**: The first case was of a 58-year-old male with advanced lung adenocarcinoma (cT4bN3M1b) with exon 18 G719X and exon 20 S781I *EGFR* mutations and received afatinib therapy. After 68 days of therapy, he developed shortness of breath and fever. Drug-induced pneumonia was not diagnosed timely, the patient received empirical antibiotics and low-dose glucocorticoids. The pulmonary inflammation rapidly progressed and the patient died 15 days after symptom onset. The second case was of a 57-year-old man with stage IV (cT3N3M1b) lung adenocarcinoma with exon 21 L861Q *EGFR* mutation. He received afatinib as second-line therapy. Fever and shortness of breath occurred 22 days after afatinib therapy, he received empirical antibiotic therapy. Five days later, CT showed aggravated pulmonary inflammation, and afatinib-induced interstitial pneumonia was diagnosed. He received glucocorticoid therapy, and the pneumonia quickly improved.

**Conclusion:** Although the incidence of EGFR-TKI-associated pneumonia is uncommon, high vigilance for drug-induced interstitial pneumonia is necessary during treatment. Early diagnosis and early glucocorticoid therapy could reverse lung injury.

## Introduction

The emergence of epidermal growth factor receptor tyrosine kinase inhibitors (EGFR-TKIs) has opened a new era in the treatment of advanced non-small cell lung cancer (NSCLC). The survival time of advanced NSCLC patients with epidermal growth factor receptor (EGFR)-activated mutation treated with EGFR-TKIs has been significantly prolonged. Further analysis of *EGFR* gene status has also shown that the rate of *EGFR*-activated mutations was high among patients with adenocarcinoma, non-smokers, East Asians, and females. Uncommon *EGFR* mutations account for about 10% of all patients with *EGFR* mutations, representing a unique and highly heterogeneous subgroup of NSCLC (O’Kane et al., 2017). The first generation EGFR-TKIs include gefitinib and erlotinib, which target the activated mutation of the *EGFR* gene. However, the efficacy of gefitinib and erlotinib in patients with uncommon *EGFR* mutations is relatively unsatisfactory. Nevertheless, second-generation EGFR-TKIs compensate for the shortage of the first-generation EGFR-TKIs in the treatment of uncommon *EGFR* mutations. Afatinib is a second-generation EGFR-TKI that irreversibly inhibits tyrosine kinase and abrogates EGFR signaling (Li et al., 2008; Solca et al., 2012). Clinical and real-world data show that afatinib is effective in patients with uncommon *EGFR* mutations (Yang et al., 2015; Yang et al., 2020). At present, afatinib is usually the first choice in clinical practice for advanced NSCLC patients with uncommon *EGFR* mutations. However, high vigilance for the occurrence of interstitial pneumonia should be practiced during clinical applications of afatinib, despite its uncommon incidence (Sharma and Graziano, 2018; Park et al., 2019). Here, we report two cases of interstitial pneumonia induced by afatinib.

## Case Description

### Case 1

**FIGURE 1:**
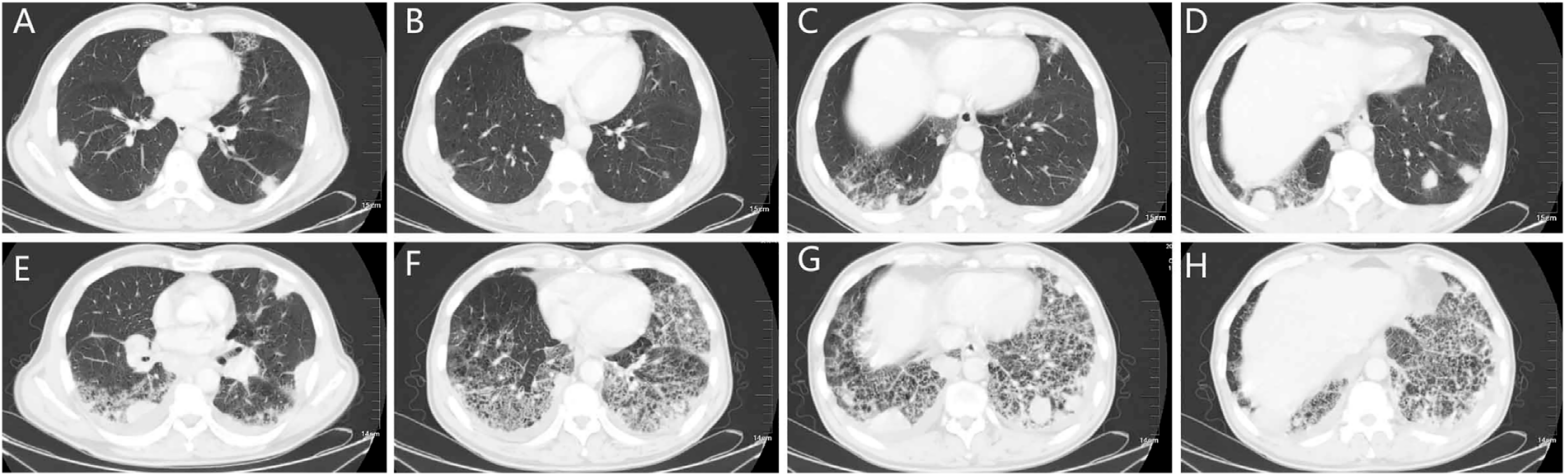
The chest CT of patient one. **(A–D)**: chest CT showing the nodes in the lungs and the local grid change in the right lower lobe. **(E–H)**: chest CT showing the pneumonitis 74 days after afatinib therapy, the pneumonitis manifested subpleural reticular shadow, ground glass and consolidation, and beehives, distributed in both lower lobe of both lungs. CT: computed tomography.

**FIGURE 2:**
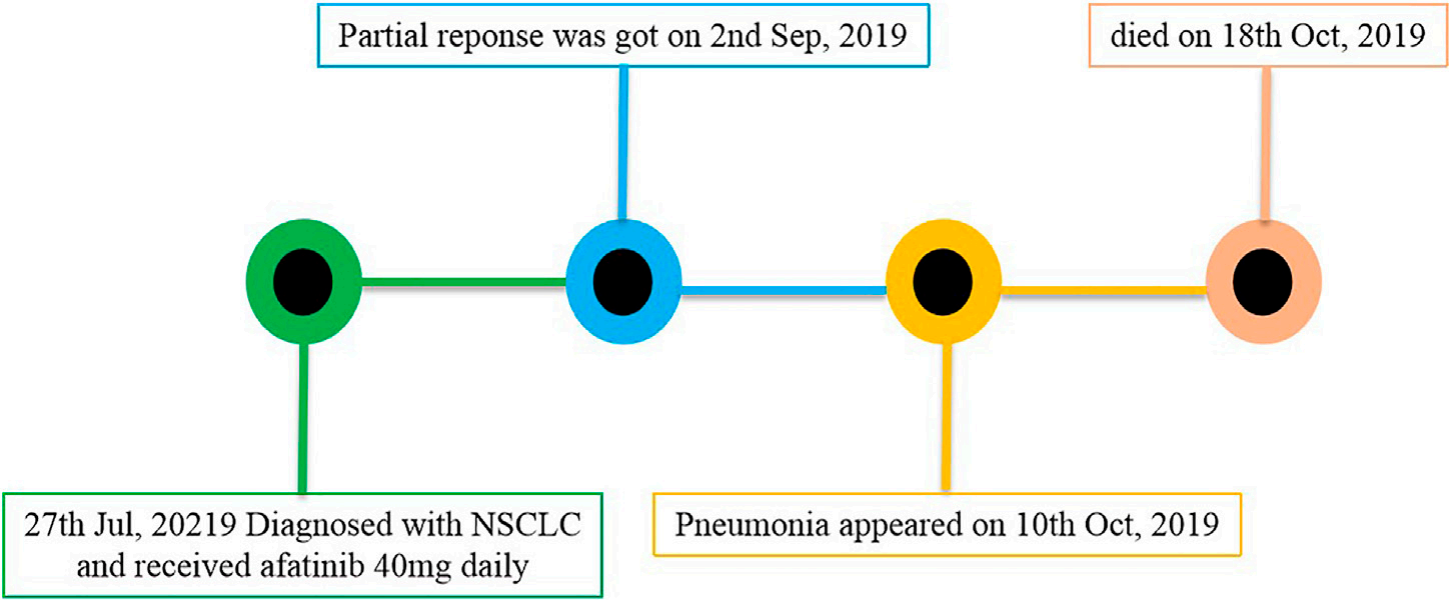
The episodes occurred in case 1.

A 58-year-old male who had no previous smoking history consulted an oncologist because of pulmonary nodules revealed by physical examination in July 2019. The diagnosis was stage IV (cT4bN3M1b) adenocarcinoma. *EGFR* mutation analysis showed mutations in exon 18 G719X and exon 20 S781I. He received 40 mg of afatinib orally every day beginning on July 27, 2019, with the treatment being well-tolerated with mild diarrhea. After 1 month, a partial response was obtained, hence the patient continued receiving afatinib therapy. However, the patient was admitted to the hospital on October 9, 2019 due to shortness of breath, fever without chills, and cough for 1 week. Figures 1A–D shows the patient’s lung lesions at partial response, and Figures 1E–H shows the lung inflammation. Physical examination showed cyanosis of the lips, short-breath, respiratory rate 26 per min, pulse 110 per min, crepitus sounds in both lungs, expecially in the lower lungs. Blood examination showed a white blood cell count of 9.7 × 10^9^/L, neutrophil percentage of 80.6%, brain natriuretic peptide (BNP) of 29 pg/ml, procalcitonin (PCT) of 0.117 ng/ml, and C-reactive protein (CRP) of 161.71 mg/L. Blood oxygen was 93% and arterial partial pressure of oxygen was 62 mmHg when admission. Serum albumin 33.2 g/L, blood urea nitrogen (BUN) 5.6 mmol/L, creatinine (Cr) 75 µmol/L, electrocardiogram showed sinus tachycardia. The glucan (G) test, galactomannan (GM) test, and sputum culture were all negative. The suspected diagnosis for the pulmonary inflammation was infection. The patient’s afatinib was discontinued and he was administered empiric antibiotics instead. At the same time, he received 40 mg of methylprednisolone daily to reduce inflammatory exudation. The patient’s shortness of breath and cough progressively worsened, and type I respiratory failure occurred 3 days later. The patient was transferred to the intensive care unit for respiratory support therapy and received broad-spectrum antibacterial therapy. The patient died from respiratory failure 9 days after admission. Figure 2 showed the episodes according to the order of occurrence for case 1.

### Case 2

**FIGURE 3:**
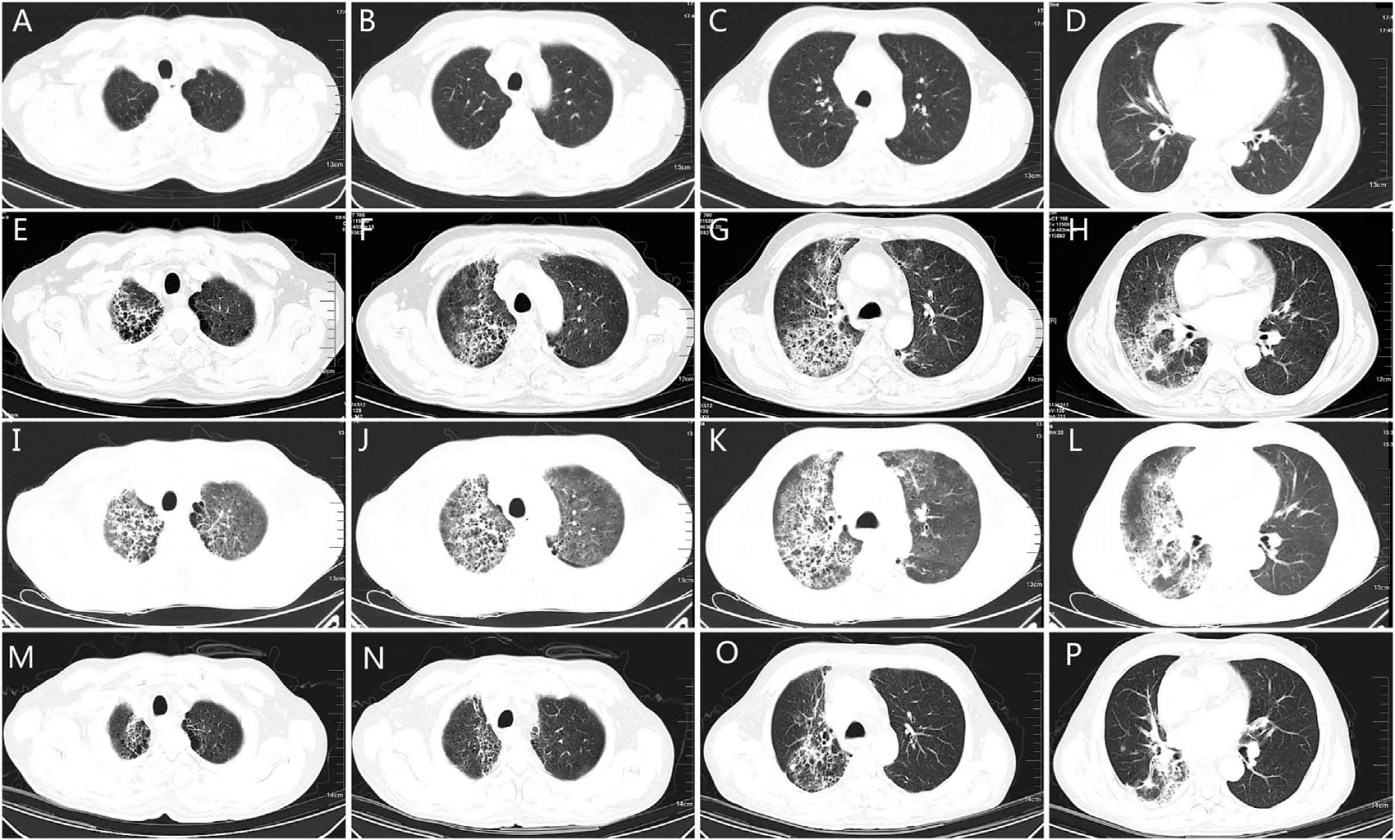
The chest CT of patient two. **(A–D)**: chest CT showing the lung quality before afatinib. **(E–H)**: chest CT showing pneumonia 27 days after afatinib therapy, the grid changes and ground glass hinting on lung injury. **(I–L)**: chest CT 5 days after discontinuation of afatinib and empirical antibiotics, showing enlarged field of grid changes and pulmonary interstitial edema, indicating the diffused alveolar injury. **(M–P)**: chest CT 17 days after glucocorticoid therapy, the grid changes and pulmonary interstitial edema reduced significantly, indicating improvements of lung injury. CT: computed tomography.

**FIGURE 4:**
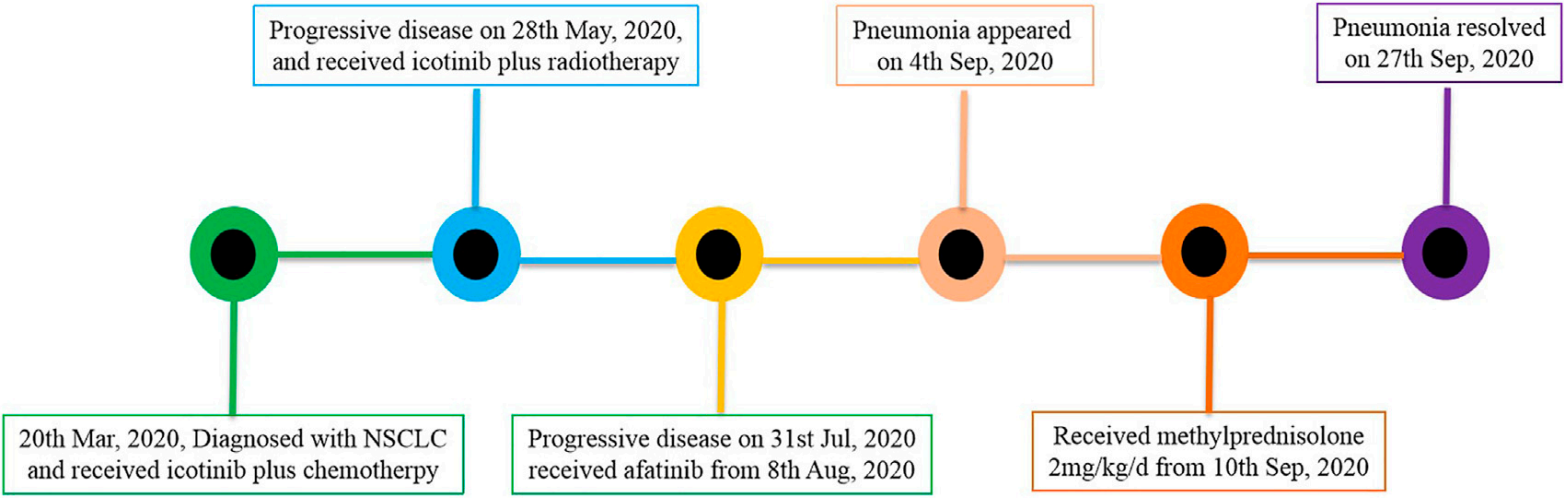
The episodes occurred in case 2.

A 57-year-old male with a smoking history of 30-pack years was diagnosed with stage IV (cT3N3M1b) adenocarcinoma in March 2020. *EGFR* mutation analysis showed an L861Q mutation in exon 21. He also had a history of atrial fibrillation. He received four cycles of icotinib plus pemetrexed and carboplatin, and experienced stable disease. However, he developed a dry cough as the mass protruded into the tracheal cavity. He received palliative radiotherapy (50Gy/25f) and simultaneous chemotherapy with pemetrexed and carboplatin for two cycles. One month later, the disease progressed with the appearance of bone and brain metastases. From August 8, 2020, he received 40 mg of afatinib daily. However, the patient developed low fever and exertional dyspnea 22 days later. Figures 3A–D showed there was no inflammation in the lungs before afatinib. Figures 3E–H showed multiple patches and grid shadows were seen on both sides of the lung. Physical examination showed cyanosis of the lips, short-breath, respiratory rate 28 per min, pulse 105 per min, crepitus sounds in both lungs, expecially in the right upper lung, and heart rate 135 per min, diastolic murmur in the tricuspid region. COVID-19 was negative by throat swab. Blood oxygen was 90% and arterial partial pressure of oxygen was 58 mmHg when admission. Blood examination revealed a white blood cell count of 4.65 × 10^9^/L, neutrophil percentage of 74.2%, BNP of 1665 pg/ml, BUN 4.6 mmol/L, Cr 51 µmol/L, PCT of 0.175 ng/ml, and CRP of 88.01 mg/L. The G test, GM test, and sputum culture were all negative. He received empiric broad-spectrum antibiotics, and afatinib was discontinued upon admission. The patient’s temperature returned to normal, but exertional dyspnea was aggravated after 1 week. CT showed a larger range of lung inflammation (Figures 3I–L). This time, a diagnosis of afatinib-induced pneumonitis was made, and the patient received methylprednisolone at the dose of 2 mg/Kg/d. Three days later, the patient experienced relief from shortness of breath. After 1 week the dose of methylprednisolone was gradually reduced, and no recurrent symptoms occurred. Figures 3M–P shows lung inflammation 17 days after the first dose of methylprednisolone. Figure 4 showed the episodes according to the order of occurrence for case 2. The cancer progressed and he was subsequently shifted to anlotinib therapy.

## Discussion

The occurrence of interstitial pneumonitis during EGFR-TKIs treatment should be monitored, despite the drugs being well-tolerated. The common adverse events associated with EGFR-TKIs are rash and diarrhea, and these adverse events are usually mild and easy to be controlled. Since the advent of these agents, severe and even fatal interstitial pneumonitis has been occasionally reported. However, the exact mechanisms of interstitial pneumonitis caused by EGFR-TKIs remain unknown. Some studies have shown that EGFR and its family members are upregulated in the early stage of acute lung injury, indicating that EGFR members might play a role in its repair (Hardie et al., 1999; Higenbottam et al., 2004). This implies that inhibition of the EGFR signaling pathway may damage lung injury repair. In clinical studies, the incidence of interstitial pneumonitis induced by afatinib is relatively low, usually occurring approximately 1 month after the initiation of therapy (Wu et al., 2014; Soria et al., 2015). In two clinical studies, no afatinib-induced interstitial pneumonitis occurred in the Chinese patient subgroup (Lu et al., 2018; Wu et al., 2018). However, in a real-world study in Japan, the incidence of afatinib-induced pneumonitis was as high as 4%. Further analysis showed that there was a high incidence of afatinib-induced pneumonitis among males, patients with poor performance status, patients with contralateral lung metastasis, and patients who have received lung radiotherapy within 1 year (Tamura et al., 2019). Both patients in the current report were males and had contralateral lung metastases. The second patient received radiotherapy within 3 months before afatinib administration. Hence, both had high risk factors for afatinib-induced interstitial pneumonitis.

During imaging, the pulmonary adverse events induced by EGFR-TKI agents may overlap with disease progression and infection, which makes it challenging for the clinician to make the correct diagnosis. The first patient in this report had a history of staying at a duck farm before he developed a fever. The inflammatory index in the patient’s blood was significantly higher than normal, and strips and patches were observed in both lungs through CT, with high possibility of pulmonary fungal infection as well as bacterial infection that could not be ruled out. The patient received empirical antibiotics for 3 days, but the treatment was ineffective. The patient’s pneumonitis rapidly progressed, and he died from respiratory failure several days later. Although this patient received glucocorticoid therapy, as the diagnosis of drug-induced interstitial pneumonitis was neglected, the glucocorticoid dosage may not have been sufficient to block further development of the pulmonary inflammation. High-dose glucocorticoids are reportedly effective in the treatment of severe interstitial pneumonitis induced by afatinib (Fujita et al., 2017), [16]; hence, it is likely that the treatment failure could be ascribed to insufficient glucocorticoid use. Afatinib-induced interstitial pneumonitis was also diagnosed after failure of empirical antibiotics in the second patient, who received high-dose glucocorticoid therapy, and hence recovered from the pneumonitis. In both patients, shortness of breath and fever were present coupled with the PCT and CRP indices and the treatment was focused on opportunistic infection (fungal infection). Both patients received empirical antibiotics. Although the incidence of EGFR-TKI-induced interstitial pneumonitis is very low, the consequences could be very serious or even fatal. For patients with dyspnea and shortness of breath who are taking EGFR-TKIs, the possibility of drug-induced interstitial pneumonitis should be assessed even if the patient has a fever and a high index of inflammation. High doses of glucocorticoids should be administered in time when drug-induced interstitial pneumonitis is suspected, and should be combined with other immunosuppressants if necessary.

## Data Availability

The original contributions presented in the study are included in the article/Supplementary Material, further inquiries can be directed to the corresponding author.

## References

[B1] Fujita K. Morishima Y. Aida Y. Tsunoda Y. Hida N. Shiozawa T. (2017). Afatinib-Induced Severe Interstitial Lung Disease Successfully Treated with High-Dose Corticosteroid Therapy: A Case Report. J. Thorac. Oncol. 12 (2), e11–13. 10.1016/j.jtho.2016.10.011 27780779

[B2] Hardie W. D. Prows D. R. Leikauf G. D. Korfhagen T. R. (1999). Attenuation of Acute Lung Injury in Transgenic Mice Expressing Human Transforming Growth Factor-Alpha. Am. J. Physiol. 277 (5), L1045–L1050. 10.1152/ajplung.1999.277.5.L1045 10564191

[B3] Higenbottam T. Kuwano K. Nemery B. Fujita Y. (2004). Understanding the Mechanisms of Drug-Associated Interstitial Lung Disease. Br. J. Cancer 91 (Suppl. 2), S31–S37. 10.1038/sj.bjc.6602065 15340376 PMC2750813

[B4] Li D. Ambrogio L. Shimamura T. Kubo S. Takahashi M. Chirieac L. R. (2008). BIBW2992, an Irreversible EGFR/HER2 Inhibitor Highly Effective in Preclinical Lung Cancer Models. Oncogene 27 (34), 4702–4711. 10.1038/onc.2008.109 18408761 PMC2748240

[B5] Lu S. Li W. Zhou C. Hu C. P. Qin S. Cheng G. (2018). Afatinib vs Erlotinib for Second-Line Treatment of Chinese Patients with Advanced Squamous Cell Carcinoma of the Lung. Onco Targets Ther. 11, 8565–8573. 10.2147/OTT.S161506 30573970 PMC6292388

[B6] O'Kane G. M. Bradbury P. A. Feld R. Leighl N. B. Liu G. Pisters K. M. (2017). Uncommon EGFR Mutations in Advanced Non-small Cell Lung Cancer. Lung Cancer 109, 137–144. 10.1016/j.lungcan.2017.04.016 28577943

[B7] Park K. Wan-Teck Lim D. Okamoto I. Yang J. C. (2019). First-line Afatinib for the Treatment of EGFR Mutation-Positive Non-small-cell Lung Cancer in the 'real-World' Clinical Setting. Ther. Adv. Med. Oncol. 11, 1758835919836374. 10.1177/1758835919836374 31019567 PMC6466470

[B8] Sharma N. Graziano S. (2018). Overview of the LUX-Lung Clinical Trial Program of Afatinib for Non-small Cell Lung Cancer. Cancer Treat. Rev. 69, 143–151. 10.1016/j.ctrv.2018.06.018 30014952

[B9] Solca F. Dahl G. Zoephel A. Bader G. Sanderson M. Klein C. (2012). Target Binding Properties and Cellular Activity of Afatinib (BIBW 2992), an Irreversible ErbB Family Blocker. J. Pharmacol. Exp. Ther. 343 (2), 342–350. 10.1124/jpet.112.197756 22888144

[B10] Soria J. C. Felip E. Cobo M. Lu S. Syrigos K. Lee K. H. (2015). Afatinib versus Erlotinib as Second-Line Treatment of Patients with Advanced Squamous Cell Carcinoma of the Lung (LUX-Lung 8): an Open-Label Randomised Controlled Phase 3 Trial. Lancet Oncol. 16 (8), 897–907. 10.1016/S1470-2045(15)00006-6 26156651

[B11] Tamura K. Nukiwa T. Gemma A. Yamamoto N. Mizushima M. Ochai K. (2019). Real-world Treatment of over 1600 Japanese Patients with EGFR Mutation-Positive Non-small Cell Lung Cancer with Daily Afatinib. Int. J. Clin. Oncol. 24 (8), 917–926. 10.1007/s10147-019-01439-5 30953238 PMC6597604

[B12] Wu Y. L. Xu C. R. Hu C. P. Feng J. Lu S. Huang Y. (2018). Afatinib versus Gemcitabine/cisplatin for First-Line Treatment of Chinese Patients with Advanced Non-small-cell Lung Cancer Harboring EGFR Mutations: Subgroup Analysis of the LUX-Lung 6 Trial. Onco Targets Ther. 11, 8575–8587. 10.2147/OTT.S160358 30584317 PMC6280988

[B13] Wu Y. L. Zhou C. Hu C. P. Feng J. Lu S. Huang Y. (2014). Afatinib versus Cisplatin Plus Gemcitabine for First-Line Treatment of Asian Patients with Advanced Non-small-cell Lung Cancer Harbouring EGFR Mutations (LUX-Lung 6): an Open-Label, Randomised Phase 3 Trial. Lancet Oncol. 15 (2), 213–222. 10.1016/S1470-2045(13)70604-1 24439929

[B14] Yang J. C. Schuler M. Popat S. Miura S. Heeke S. Park K. (2020). Afatinib for the Treatment of NSCLC Harboring Uncommon EGFR Mutations: A Database of 693 Cases. J. Thorac. Oncol. 15 (5), 803–815. 10.1016/j.jtho.2019.12.126 31931137

[B15] Yang J. C. Sequist L. V. Geater S. L. Tsai C. M. Mok T. S. Schuler M. (2015). Clinical Activity of Afatinib in Patients with Advanced Non-small-cell Lung Cancer Harbouring Uncommon EGFR Mutations: a Combined post-hoc Analysis of LUX-Lung 2, LUX-Lung 3, and LUX-Lung 6. Lancet Oncol. 16 (7), 830–838. 10.1016/S1470-2045(15)00026-1 26051236

